# Supplementary material for: Cellular Mechanism Underlying Highly-Active or Antiretroviral Therapy-Induced Lipodystrophy: Atazanavir, a Protease Inhibitor, Compromises Adipogenic Conversion of Adipose-Derived Stem/Progenitor Cells through Accelerating ER Stress-Mediated Cell Death in Differentiating Adipocytes
Source: Int J Mol Sci. 2021 Feb 20;22(4):2114. doi: 10.3390/ijms22042114 (PMC7924614; doi:10.3390/ijms22042114)
Supplement: Supplementary file 1 [file ijms-22-02114-s001.zip › ijms-1097992-supplementary.pptx]

## Slide 1
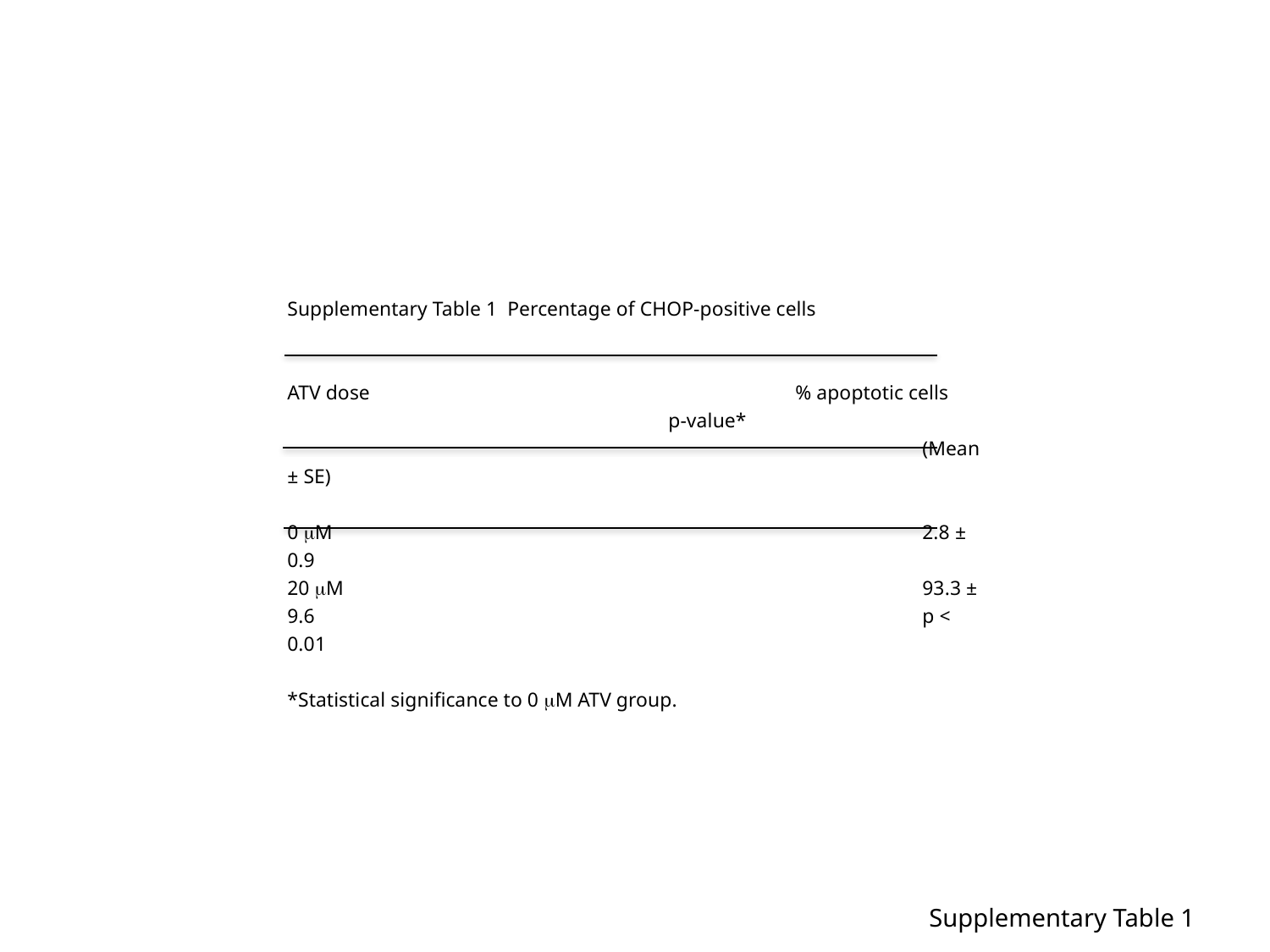

Supplementary Table 1 Percentage of CHOP-positive cells
ATV dose				% apoptotic cells			p-value*
					(Mean ± SE)
0 μM					2.8 ± 0.9
20 μM					93.3 ± 9.6					p < 0.01
*Statistical significance to 0 μM ATV group.
Supplementary Table 1

## Slide 2
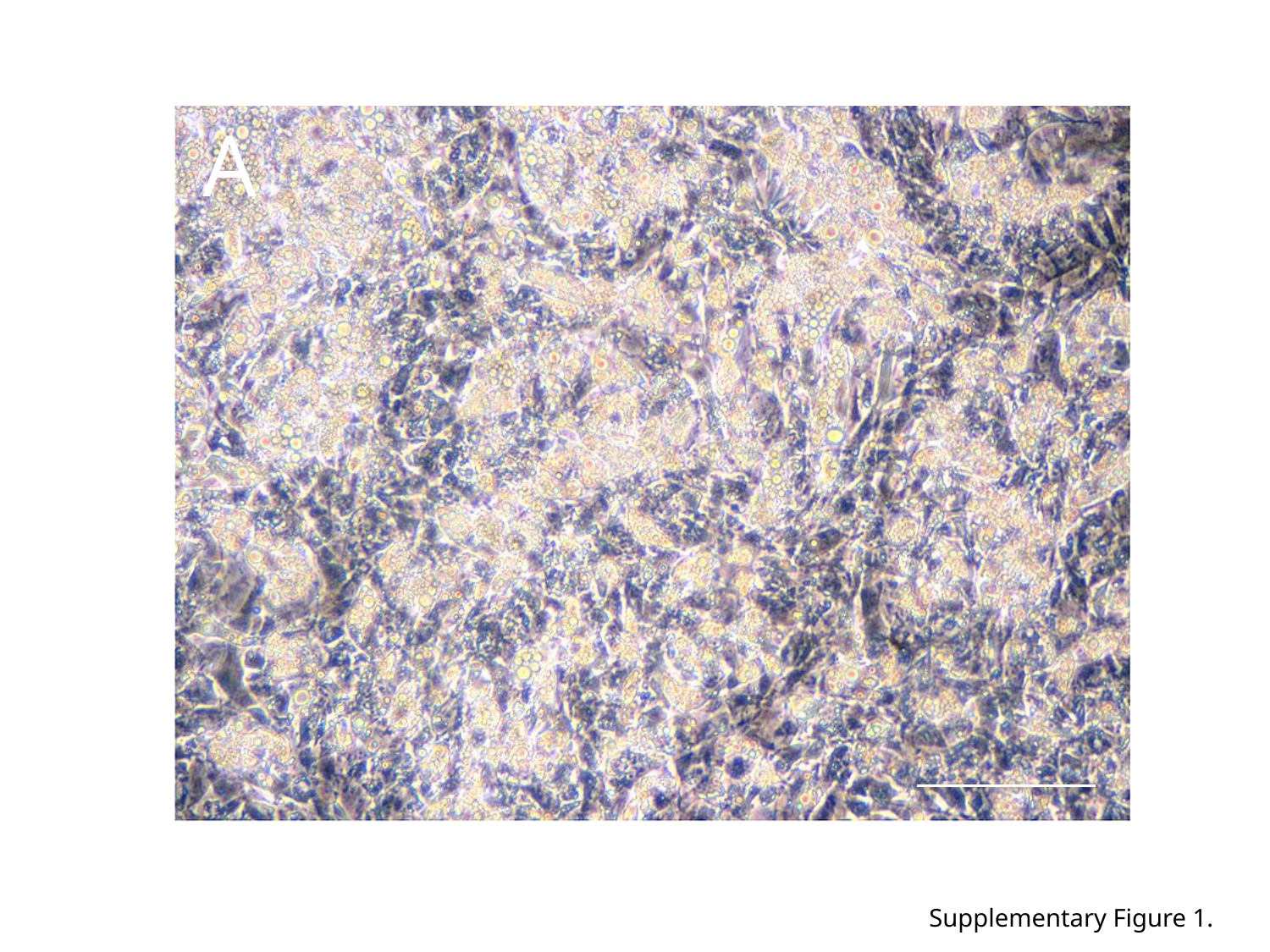

A
Supplementary Figure 1.

## Slide 3
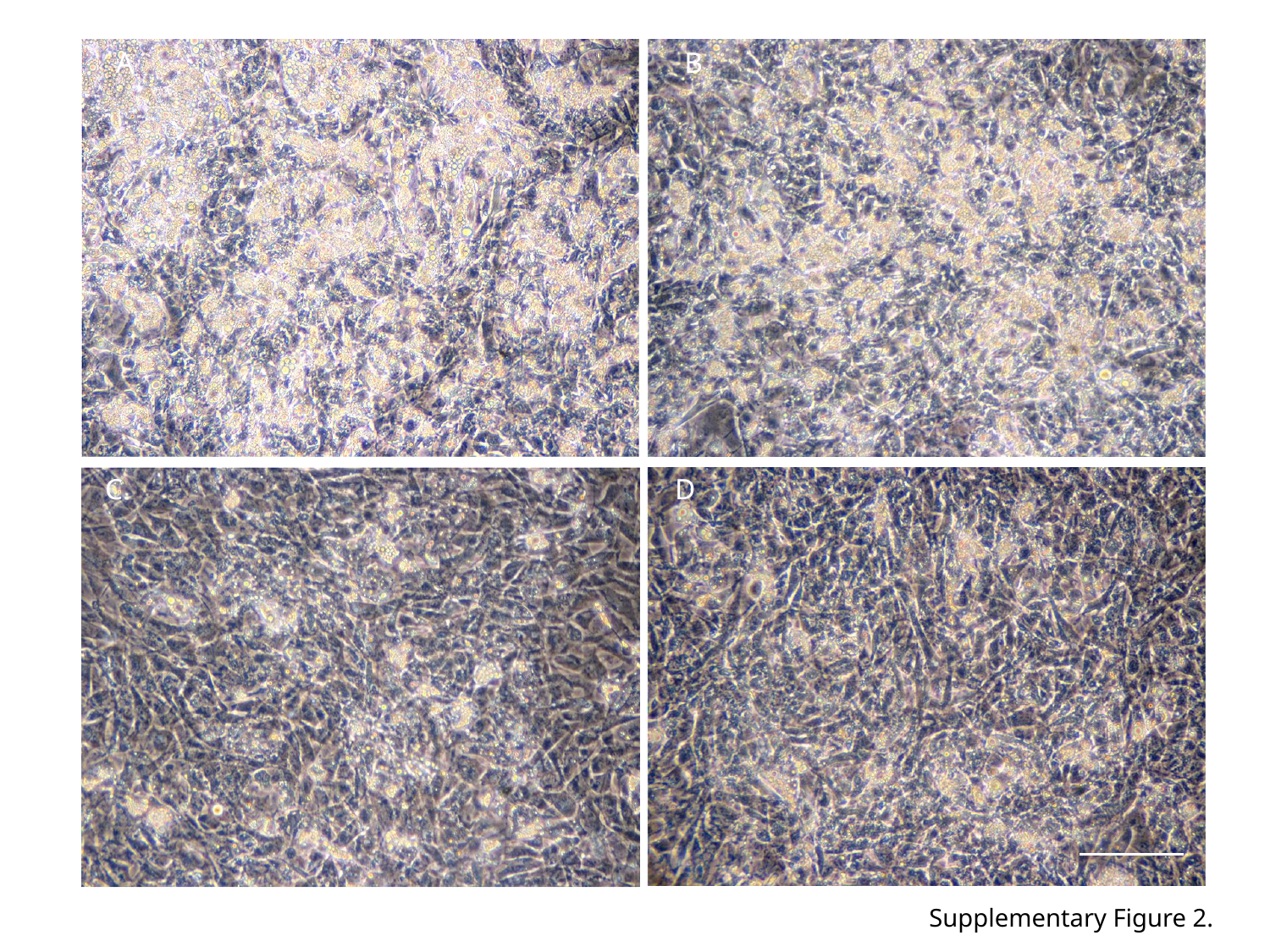

A
B
C
D
A
Supplementary Figure 2.

## Slide 4
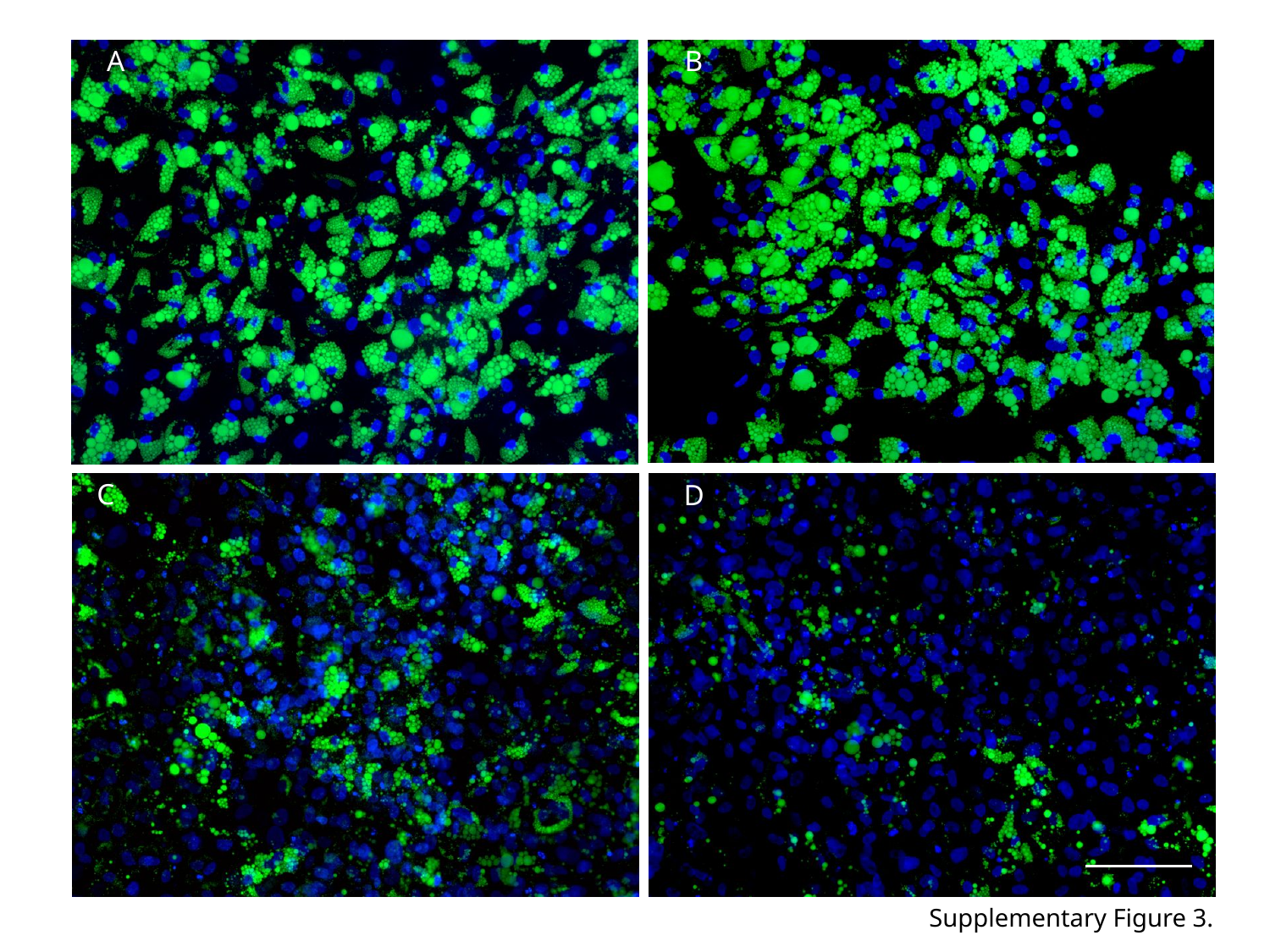

A
B
C
D
A
Supplementary Figure 3.

## Slide 5
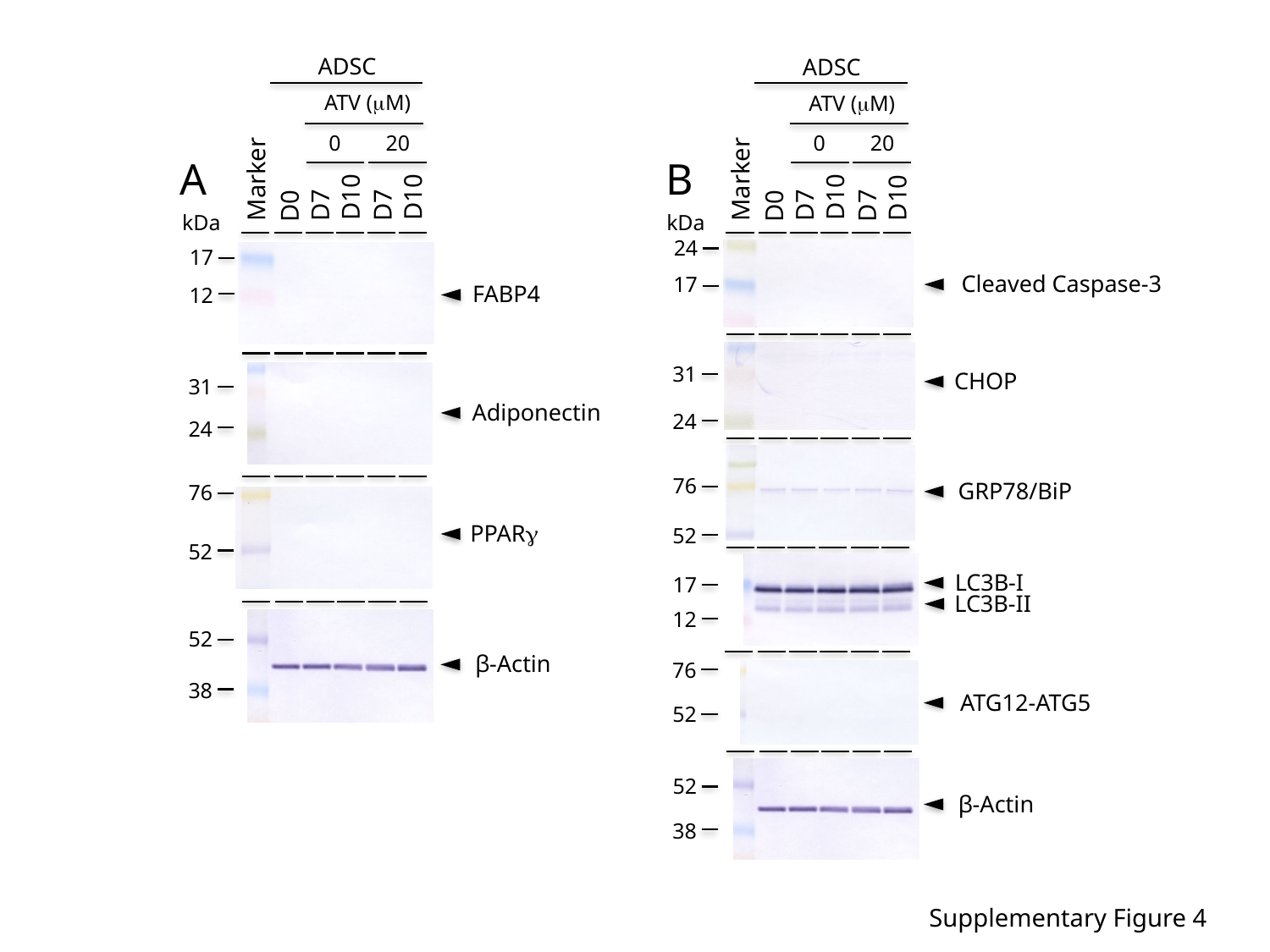

ADSC
ADSC
ATV (mM)
ATV (mM)
0
20
0
20
A
B
Marker
Marker
D10
D10
D10
D10
D7
D7
D7
D7
D0
D0
kDa
kDa
24
17
Cleaved Caspase-3
17
FABP4
12
31
CHOP
31
Adiponectin
24
24
76
GRP78/BiP
76
PPARg
52
52
LC3B-I
17
LC3B-II
12
52
β-Actin
76
38
ATG12-ATG5
52
52
β-Actin
38
Supplementary Figure 4

## Slide 6
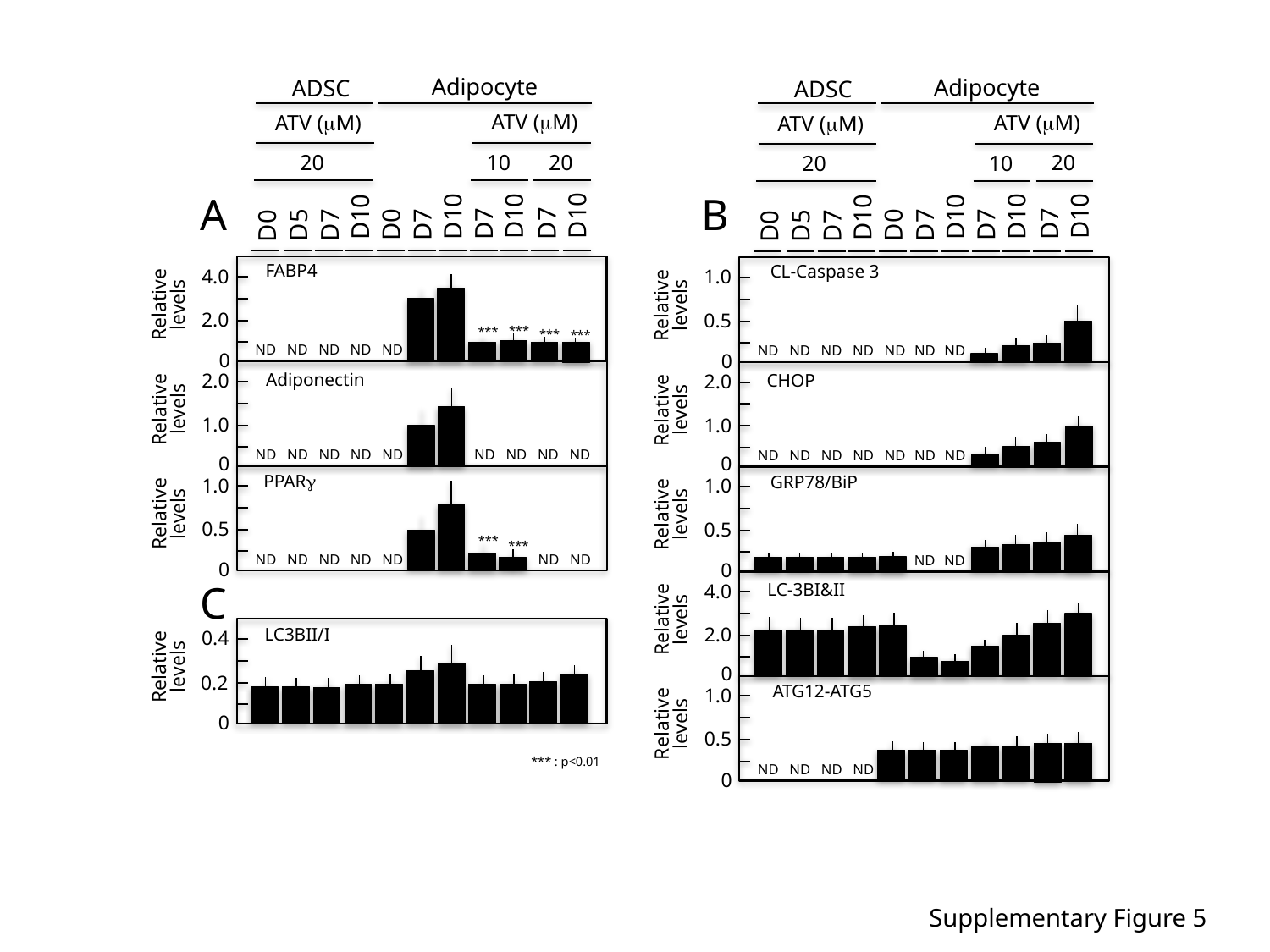

Adipocyte
Adipocyte
ADSC
ADSC
ATV (mM)
ATV (mM)
ATV (mM)
ATV (mM)
20
10
20
20
10
20
A
B
D10
D10
D10
D10
D10
D10
D10
D10
D7
D7
D7
D0
D7
D7
D0
D7
D5
D7
D5
D7
D0
D0
FABP4
4.0
CL-Caspase 3
1.0
Relative
levels
Relative
levels
2.0
0.5
***
***
***
***
ND
ND
ND
ND
ND
ND
ND
ND
ND
ND
ND
ND
0
0
2.0
2.0
Adiponectin
CHOP
Relative
levels
Relative
levels
1.0
1.0
ND
ND
ND
ND
ND
ND
ND
ND
ND
ND
ND
ND
ND
ND
ND
ND
0
0
1.0
1.0
PPARg
GRP78/BiP
Relative
levels
Relative
levels
0.5
0.5
***
***
ND
ND
ND
ND
ND
ND
ND
ND
ND
0
0
C
4.0
LC-3BI&II
Relative
levels
2.0
0.4
LC3BII/I
Relative
levels
0
0.2
1.0
ATG12-ATG5
Relative
levels
0
0.5
*** : p<0.01
ND
ND
ND
ND
0
Supplementary Figure 5

## Slide 7
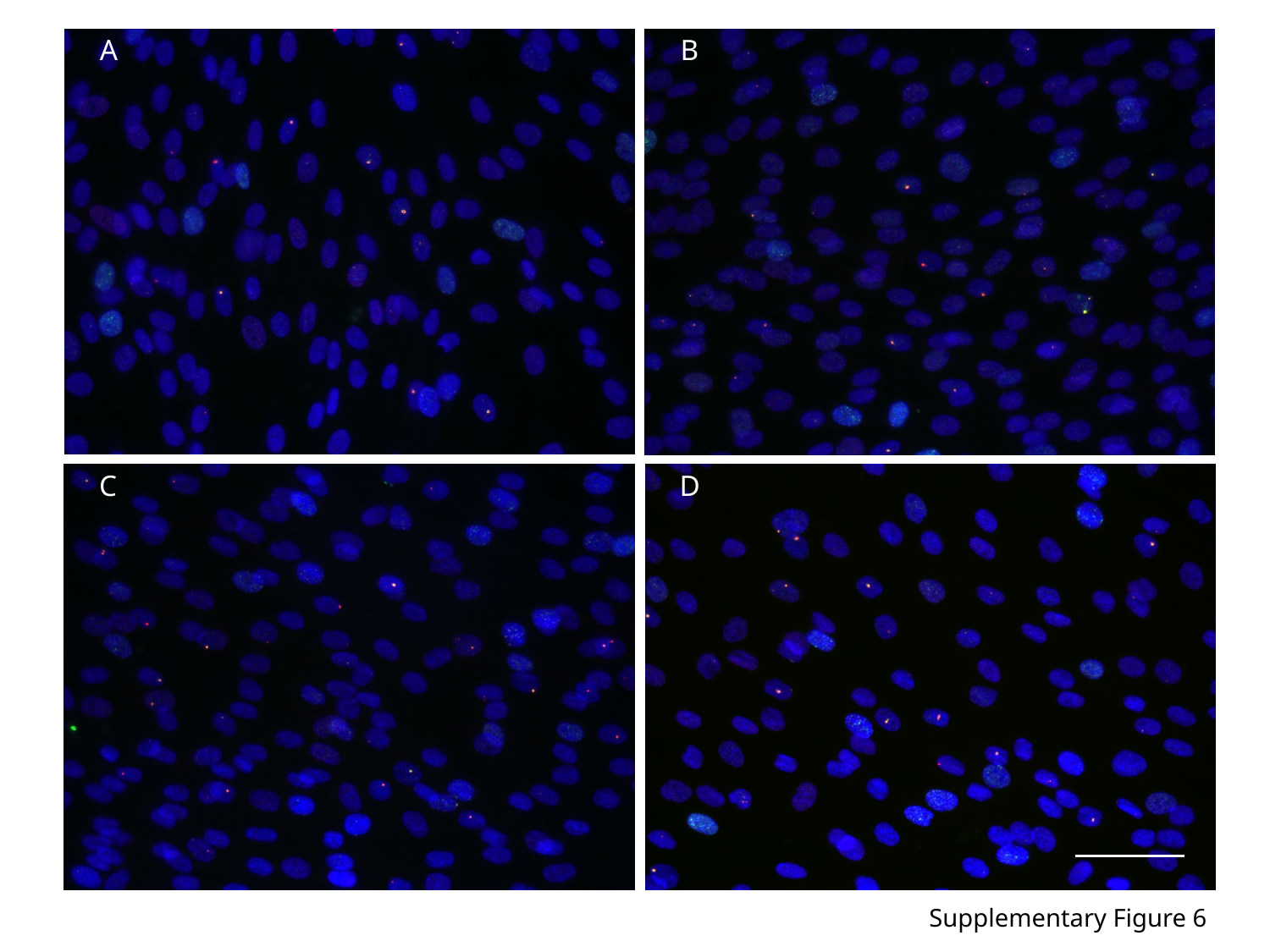

A
B
C
D
A
Supplementary Figure 6

## Slide 8
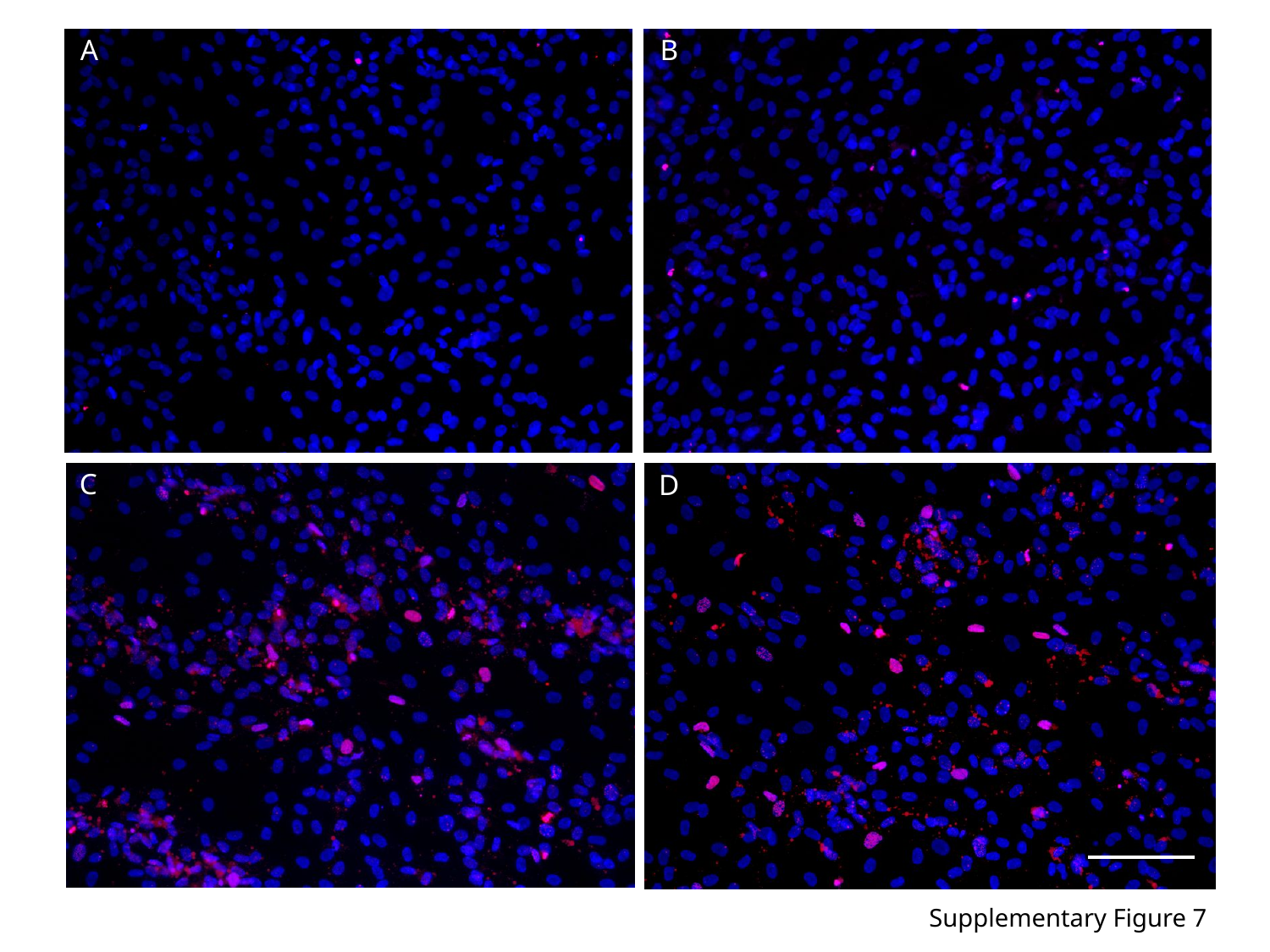

A
B
C
D
Supplementary Figure 7

## Slide 9
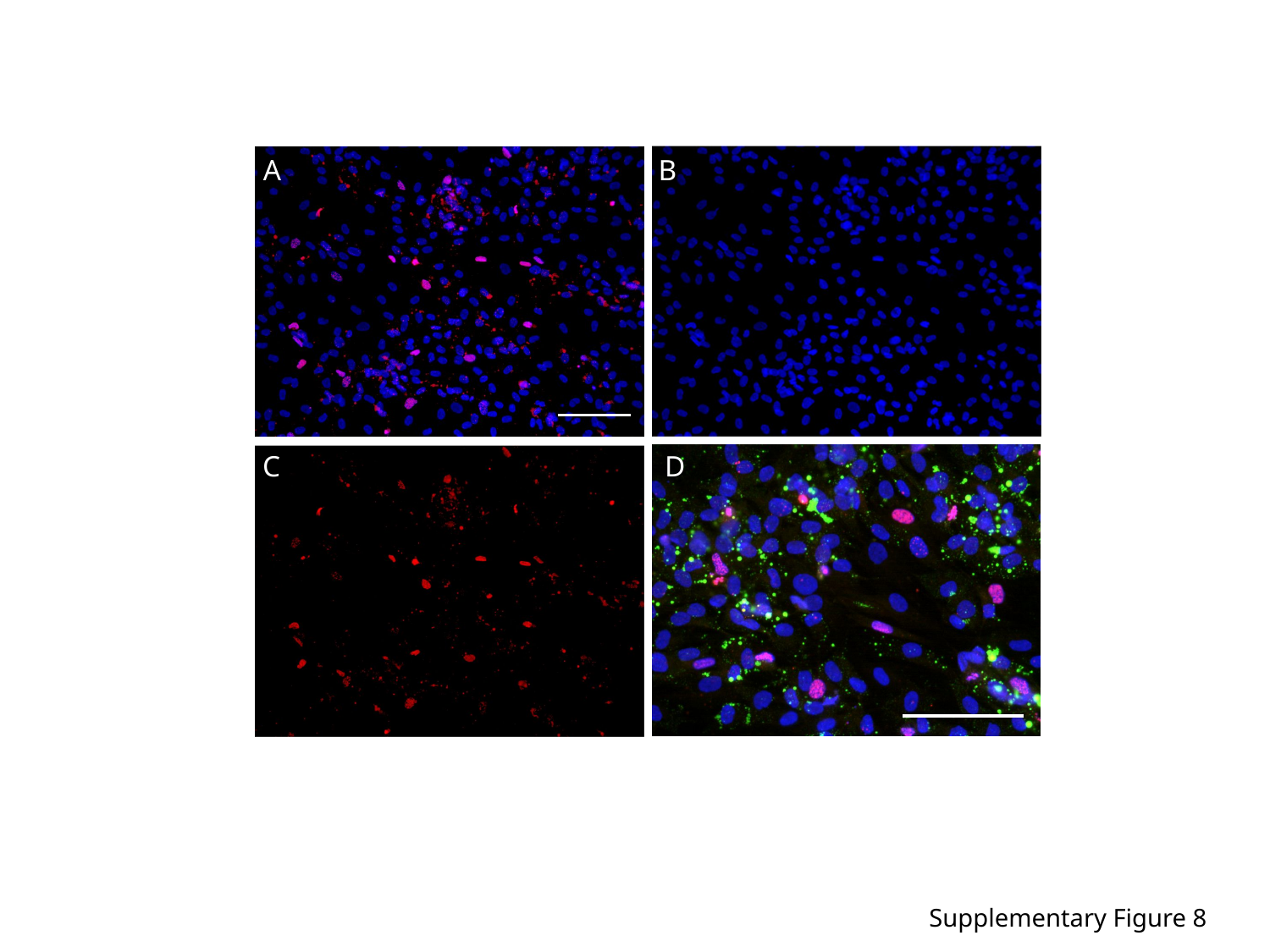

A
B
C
D
D
Supplementary Figure 8

## Slide 10
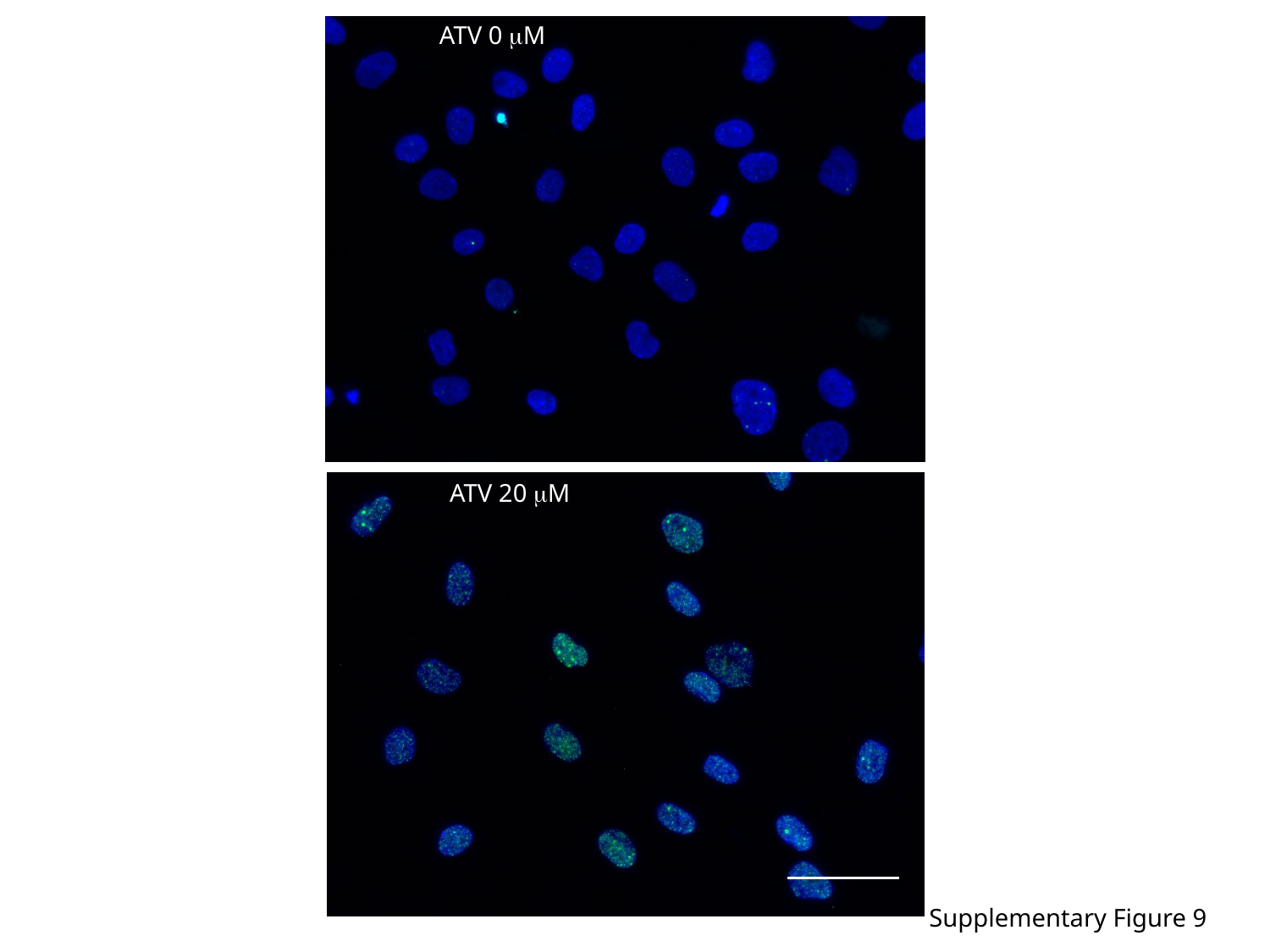

ATV 0 mM
ATV 20 mM
Supplementary Figure 9

## Slide 11
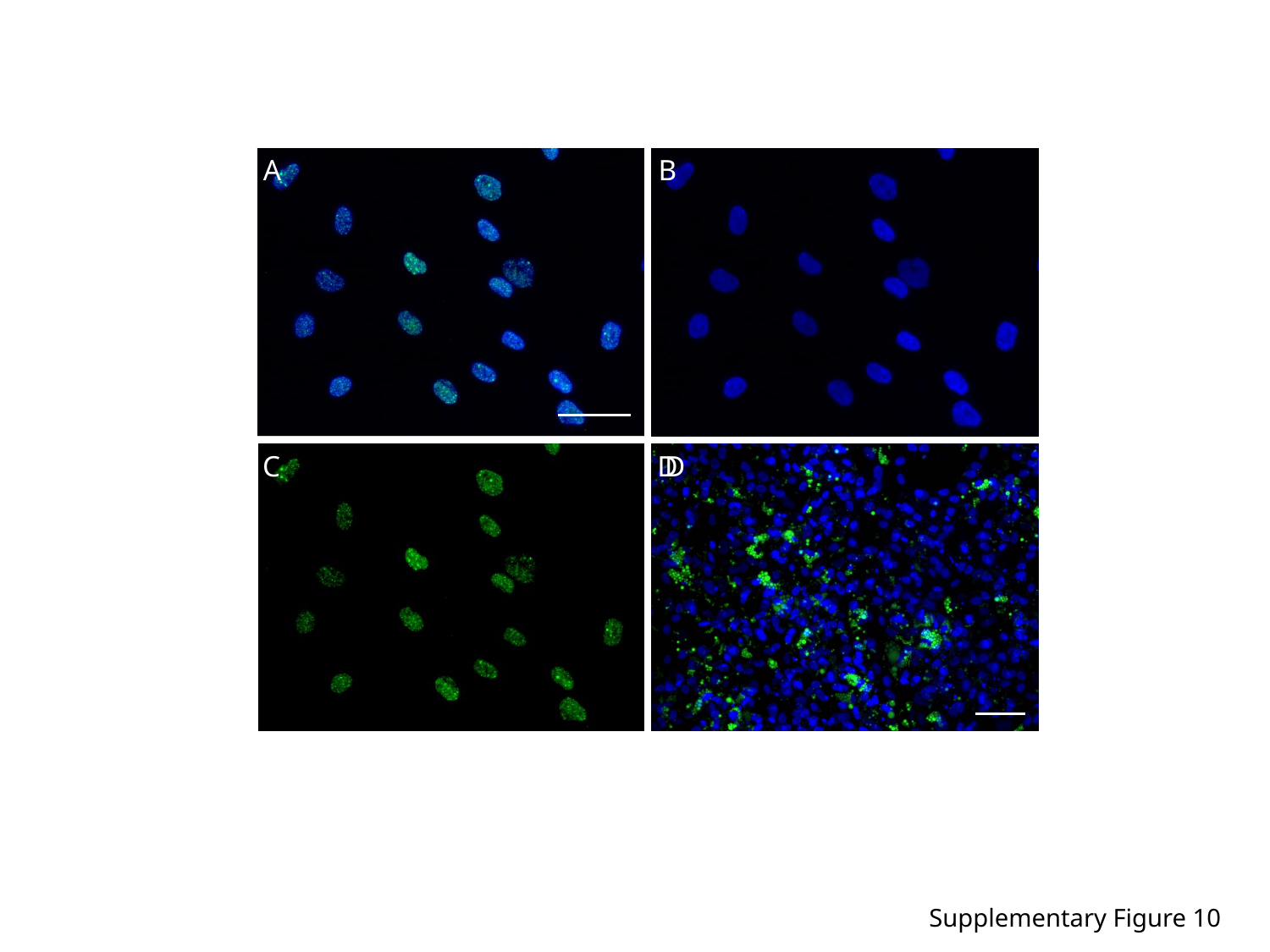

A
B
D
C
D
Supplementary Figure 10

## Slide 12
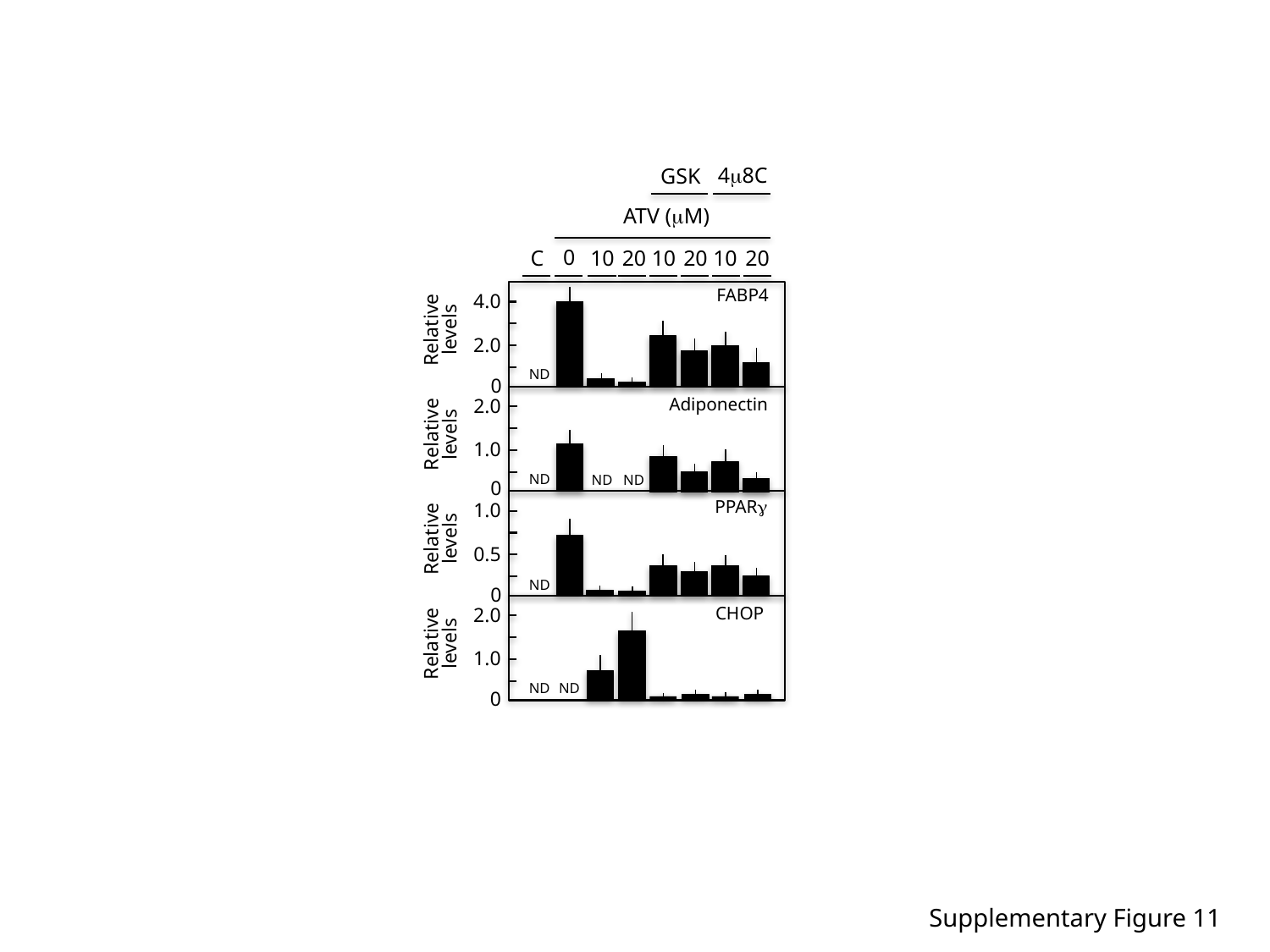

4m8C
GSK
ATV (mM)
0
C
10
20
10
20
10
20
FABP4
4.0
Relative
levels
2.0
ND
0
2.0
Adiponectin
Relative
levels
1.0
ND
ND
ND
0
1.0
PPARg
Relative
levels
0.5
ND
0
2.0
CHOP
Relative
levels
1.0
ND
ND
0
Supplementary Figure 11

## Slide 13
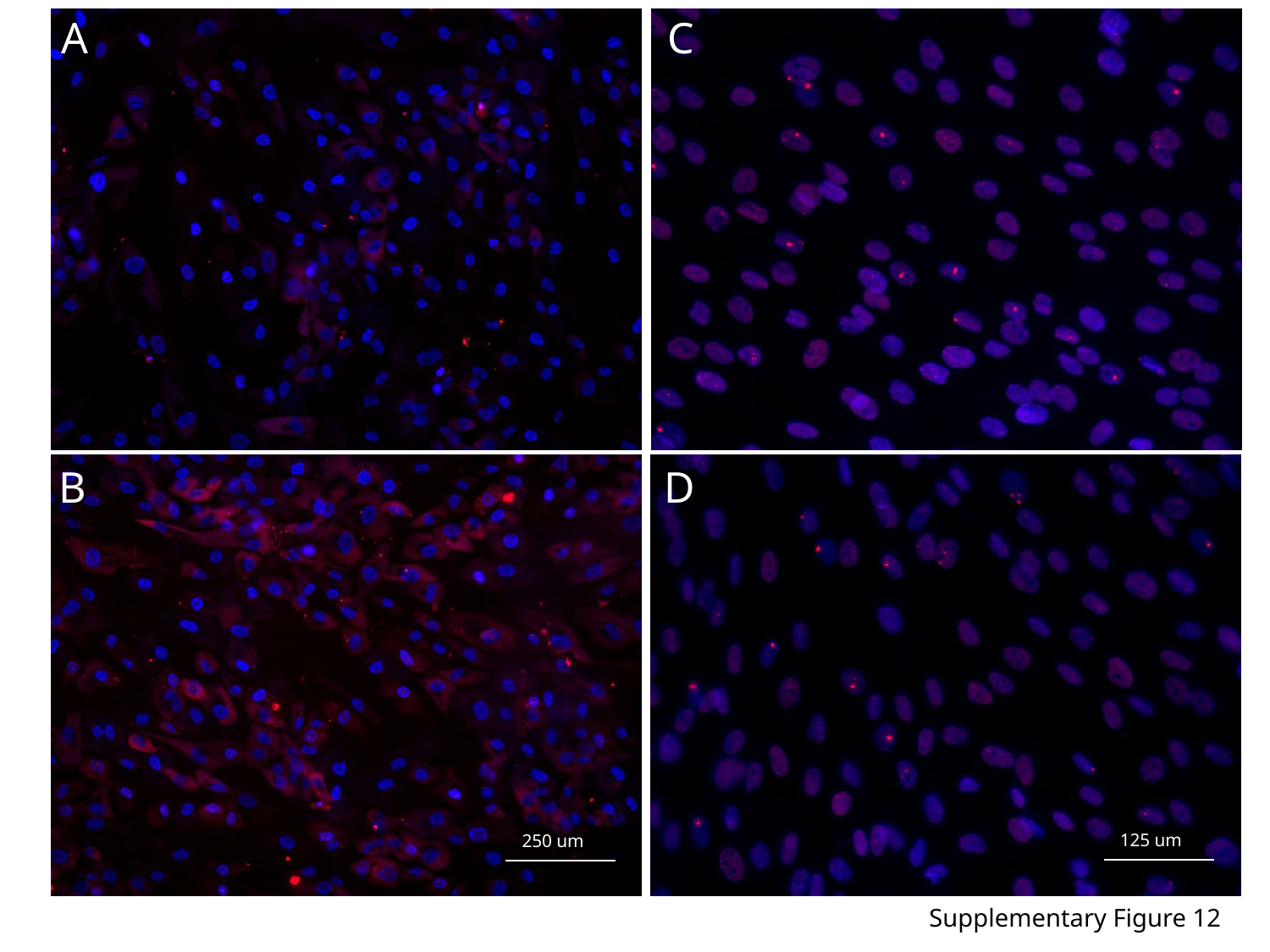

A
C
B
D
125 um
250 um
Supplementary Figure 12

## Slide 14
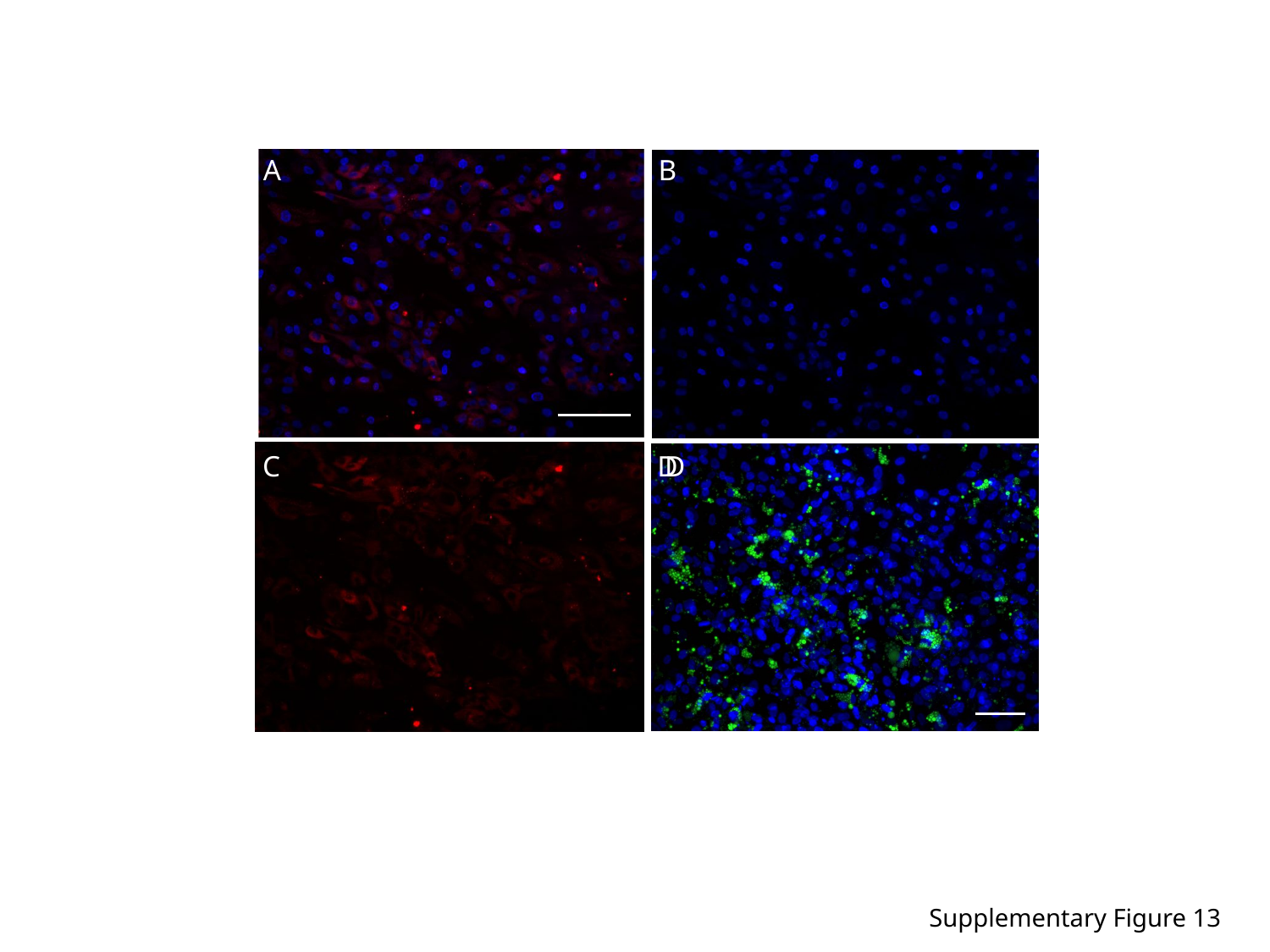

A
B
D
C
D
Supplementary Figure 13

## Slide 15
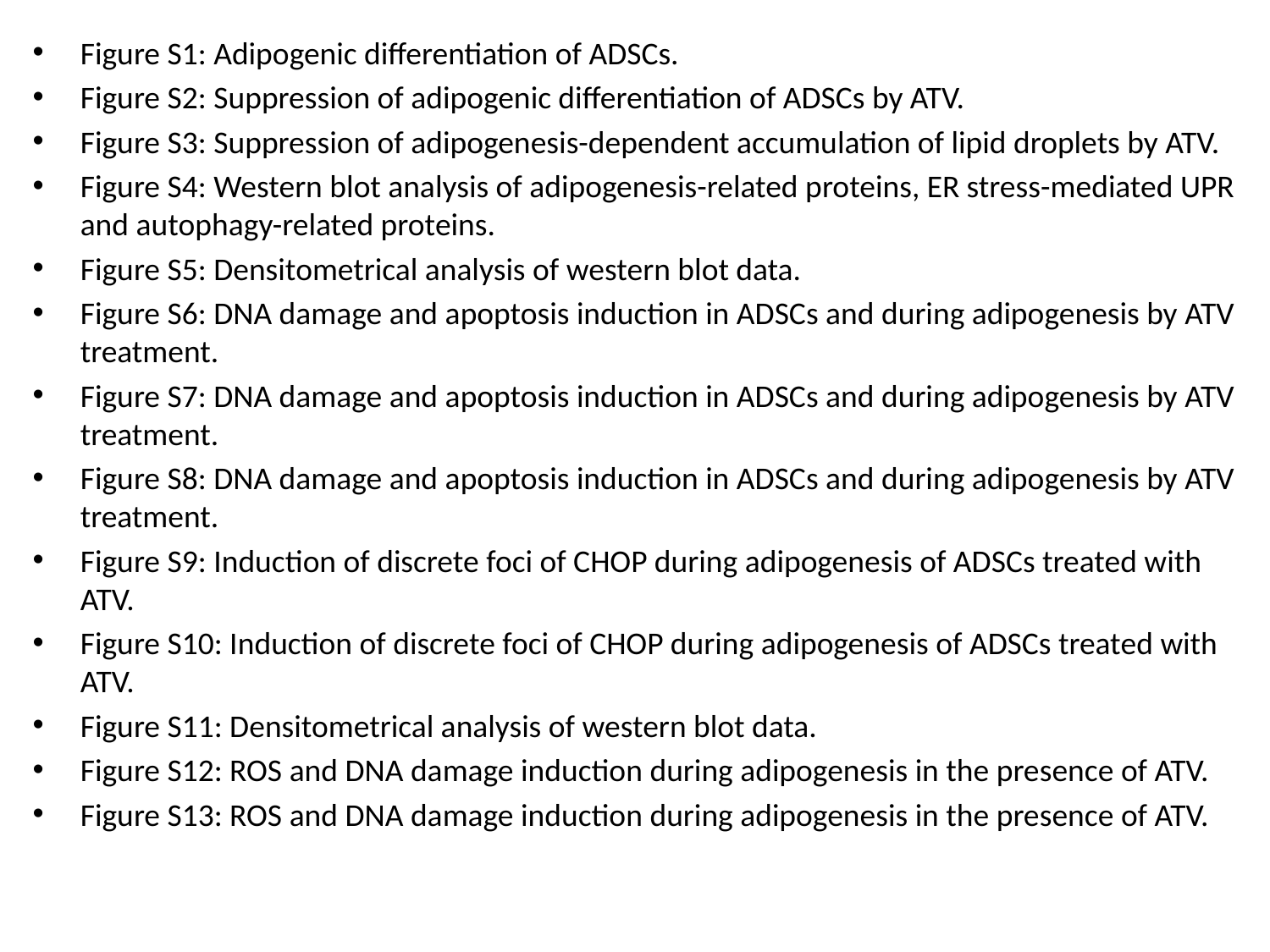

Figure S1: Adipogenic differentiation of ADSCs.
Figure S2: Suppression of adipogenic differentiation of ADSCs by ATV.
Figure S3: Suppression of adipogenesis-dependent accumulation of lipid droplets by ATV.
Figure S4: Western blot analysis of adipogenesis-related proteins, ER stress-mediated UPR and autophagy-related proteins.
Figure S5: Densitometrical analysis of western blot data.
Figure S6: DNA damage and apoptosis induction in ADSCs and during adipogenesis by ATV treatment.
Figure S7: DNA damage and apoptosis induction in ADSCs and during adipogenesis by ATV treatment.
Figure S8: DNA damage and apoptosis induction in ADSCs and during adipogenesis by ATV treatment.
Figure S9: Induction of discrete foci of CHOP during adipogenesis of ADSCs treated with ATV.
Figure S10: Induction of discrete foci of CHOP during adipogenesis of ADSCs treated with ATV.
Figure S11: Densitometrical analysis of western blot data.
Figure S12: ROS and DNA damage induction during adipogenesis in the presence of ATV.
Figure S13: ROS and DNA damage induction during adipogenesis in the presence of ATV.
